# Baseline nocturnal glucose change: A predictor of the treatment effect of bolus intensification in insulin‐treated type 2 diabetes

**DOI:** 10.1111/dom.13729

**Published:** 2019-04-23

**Authors:** Anne L. Peters, Milivoj Piletič, Johan Ejstrud, Karen Salvesen‐Sykes, James Snyder, Keith Bowering

**Affiliations:** ^1^ Clinical Diabetes Program, Keck School of Medicine of the University of Southern California Los Angeles California; ^2^ General Hospital Novo Mesto Slovenia; ^3^ Novo Nordisk A/S Aalborg Denmark; ^4^ Novo Nordisk Inc. Plainsboro New Jersey; ^5^ Division of Endocrinology and Metabolism University of Alberta Edmonton Alberta Canada

**Keywords:** clinical trial, insulin therapy, randomized trial, type 2 diabetes

## Abstract

This post hoc analysis of an 18‐week randomized trial explored the utility of calculating baseline glycated haemoglobin (HbA1c), postprandial glucose (PPG) increments and nocturnal glucose change in predicting efficacy and safety outcomes in response to bolus insulin intensification in people with type 2 diabetes (T2D). Analyses were conducted on 236 participants with T2D receiving metformin: 116 received fast‐acting insulin aspart (faster aspart) basal–bolus therapy and 120 received basal‐only insulin. Participants were grouped according to baseline HbA1c, PPG increments and nocturnal glucose change variables; analyses were performed on the end‐of‐trial treatment differences between “high” and “low” baseline values. The change from baseline in end‐of‐trial mean HbA1c and mean PPG increments was in favour of faster aspart across all subgroups. Significantly greater treatment differences were observed in participants with high (vs. low) baseline nocturnal glucose change and PPG increments. For baseline HbA1c, significantly greater treatment differences were observed for change in end‐of‐trial PPG increments, but not end‐of‐trial HbA1c. In conclusion, both nocturnal glucose change and PPG increments may be more useful than HbA1c for identifying subgroups of people with T2D who would most benefit from bolus intensification.

## 1 INTRODUCTION

In people with type 2 diabetes (T2D), suboptimal glycaemic control increases the risk of developing micro‐ and macrovascular complications.^1^ For non‐pregnant adults, current guidelines recommend a glycated haemoglobin (HbA1c) target of <53 mmol/mol (7.0%).^2^ When patients do not achieve glycaemic targets using a combination of lifestyle modifications and oral antidiabetic drugs, treatment can be intensified with basal insulin,^2^ but this alone is not always effective.^3^ As HbA1c approaches 53 mmol/mol (7.0%), postprandial glucose (PPG), rather than fasting plasma glucose, becomes the dominant contributor to overall HbA1c, and many people require further treatment intensification with drugs targeting PPG excursions (eg, mealtime bolus insulin).^2^, ^4^, ^5^ In routine clinical practice, it is difficult to identify, in a timely manner, the people with T2D who would most likely benefit from intensification with bolus insulin,^6^ which may result in the uptitration of basal insulin, increasing risk of weight gain and hypoglycaemia.^7^

Characteristics related to severity of disease, such as elevated HbA1c and self‐measured blood glucose (SMBG)‐derived PPG increments, are associated with suboptimal glycaemic control in people with T2D and have been shown to predict the HbA1c‐lowering effect of insulin therapy.^5^, ^8^, ^9^ However, HbA1c does not provide an indication of short‐term glycaemic control, and measuring PPG increments can be time‐consuming and disrupt patients' lives.^10^ The change in nocturnal blood glucose from bedtime to pre‐breakfast (termed herein as “nocturnal glucose change”) is an SMBG‐derived measure that only requires two blood glucose readings; it is easier and quicker to calculate than PPG increments and, when elevated, indicates a need to target PPG.^11^ Moreover, calculating baseline nocturnal glucose change may provide a simple method for identifying patients who would benefit most from bolus insulin intensification.

To evaluate the value of calculating baseline nocturnal glucose change compared with PPG increments, existing clinical trial data were used. This post hoc analysis explored the utility of HbA1c, PPG increments and nocturnal glucose change, all at baseline, in predicting efficacy and safety outcomes in participants who intensified basal insulin treatment with mealtime faster aspart.

## 2 METHODS

The present study was a post hoc analysis of the onset 3 trial (ClinicalTrials.gov: NCT01850615), an 18‐week, multicentre (51 sites across six countries), open‐label, randomized trial that compared the efficacy and safety of fast‐acting insulin aspart (faster aspart) intensification in a basal–bolus regimen (n = 116) versus continued basal‐only insulin (n = 120), both in combination with metformin.^12^ The objective of this trial was to confirm superiority of mealtime faster aspart (basal–bolus therapy) versus basal‐only therapy in terms of glycaemic control. The trial was conducted in accordance with the Declaration of Helsinki and the International Conference on Harmonization Good Clinical Practice. The full methodology and results of onset 3 have been previously reported.^12^ The trial design and inclusion criteria are summarized in Figure S1 and Appendix S1. Baseline characteristics were well matched across both treatments (Table S1 and Appendix S1). All participants were included in the post hoc analysis.

### 2.1 Post hoc analysis population and outcomes

In this post hoc analysis, we explored the predictive utility of baseline glycaemic variables (HbA1c, PPG increments and nocturnal glucose change) on clinical outcomes in T2D. Clinical outcomes included in this analysis were change from baseline in HbA1c and mean PPG increments at the visit 18 weeks post‐randomization and the rates of treatment‐emergent severe or blood glucose‐confirmed hypoglycaemia. “Treatment‐emergent” was defined as an event that had an onset up to 1 day after the last day of randomized treatment, but excluded any events that occurred during the run‐in period. Severe or blood glucose‐confirmed hypoglycaemic episodes were defined with a plasma glucose value <3.1 mmol/L (56.0 mg/dL), with or without symptoms consistent with severe hypoglycaemia.

To explore the predictive utility of baseline HbA1c, PPG increments and nocturnal glucose change, analyses were performed on the treatment outcome differences (faster aspart basal–bolus minus basal‐only therapy) at week 18, comparing “high” and “low” baseline values of each variable. “High” and “low” were defined as being above or below the median value at baseline, respectively. The median values for each variable at baseline were: 61.7 mmol/mol (7.8%) for HbA1c; 2.42 mmol/L (43.6 mg/dL) for PPG increment; and 3.11 mmol/L (56 mg/dL) for nocturnal glucose change. The correlation between these variables at baseline was also investigated.

The PPG increments were calculated as the difference between SMBG readings 2 hours post‐meal and pre‐meal, and these were recorded from seven‐point profiles over a 3‐day period, and averaged over time. Nocturnal glucose change was calculated as the difference between SMBG at bedtime and pre‐breakfast the following day. From seven‐point SMBG profiles over a 3‐day period, two values could be calculated with a complete dataset, which were averaged to calculate mean nocturnal glucose change.

Definitions of the analysis sets, and details of the statistical models and tests, are provided in Appendix S1.

## 3 RESULTS

### 3.1 Correlation of HbA1c, mean PPG increment and nocturnal glucose change at baseline

At baseline, there was a positive correlation between PPG increment and nocturnal glucose change (r = 0.66) and between HbA1c and nocturnal glucose change (r = 0.20). There was a small correlation between PPG increment and HbA1c (r = 0.11).

### 3.2 Association between baseline characteristics and end‐of‐trial HbA1c and PPG

**Figure 1:**
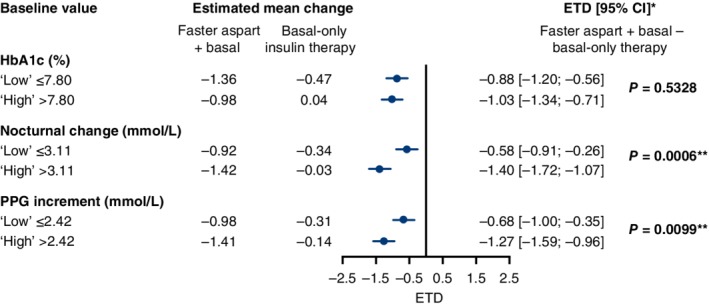
Change from baseline in glycated haemoglobin (HbA1c) at week 18, stratified by baseline variables. *Negative values are in favour of fast‐acting insulin aspart (faster aspart). **Statistically significant difference between estimated treatment difference (ETD) reported in the “high” and “low” subgroups. CI, confidence interval; PPG, postprandial glucose

**Figure 2:**
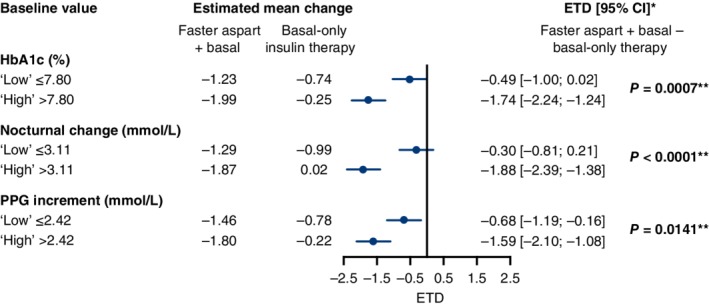
Change from baseline in mean postprandial glucose (PPG) increment at week 18, stratified by baseline variables. *Negative values are in favour of fast‐acting insulin aspart (faster aspart). **Statistically significant difference between estimated treatment difference (ETD) reported in “high” and “low” subgroups. CI, confidence interval; HbA1c, glycated haemoglobin

Changes in HbA1c and mean PPG increment from baseline to week 18, stratified by baseline variables, are shown in Figures 1 and 2, respectively. Treatment differences in these outcomes were consistently in favour of faster aspart basal–bolus therapy versus basal‐only therapy in all of the “high” and “low” baseline subgroups. There were statistically significantly greater treatment differences in change in HbA1c in participants with “high” baseline nocturnal glucose change (*P* = 0.0006) and PPG increment (*P* = 0.0099) than in those with “low” values. There was no statistically significant treatment difference in change in HbA1c (*P* = 0.533) between participants with “high” and “low” baseline HbA1c values (Figure 1). Statistically significantly greater treatment differences in the change from baseline in mean PPG increment were present in participants with “high” baseline HbA1c (*P* = 0.0007)_,_ nocturnal glucose change (*P* < 0.0001) and PPG increment (*P* = 0.0141) than those with “low” baseline values (Figure 2).

### 3.3 Association between baseline characteristics and hypoglycaemic episodes

Overall, there were higher absolute rates of severe or blood glucose‐confirmed hypoglycaemia in those participants receiving faster aspart basal–bolus therapy versus basal‐only therapy (Figure S2). The absolute rates of hypoglycaemia were numerically greater in both treatment arms in those with “high” versus “low” baseline HbA1c, PPG increment and nocturnal glucose change values; however, there were no statistically significant differences in treatment rate ratios of severe or blood glucose‐confirmed hypoglycaemia between the “high” and “low” baseline subgroups (Figure S2).

## 4 DISCUSSION

In this post hoc analysis of the onset 3 trial, participants with T2D who had a baseline PPG increment measurement >2.42 mmol/L (43.6 mg/dL) and nocturnal glucose change measurement >3.11 mmol/L (56 mg/dL) experienced greater benefit, in terms of reduction in HbA1c and PPG increment at end‐of‐trial, with basal–bolus therapy versus basal‐only therapy compared with those participants with measurements under these cut‐off values. Those with baseline HbA1c >61.7 mmol/mol (7.8%) also experienced greater benefit from basal–bolus therapy, but only in terms of reduction in PPG increment. These findings suggest, in this study population, that baseline values of nocturnal glucose change and PPG increment may be better predictors for response to intensification with bolus insulin than baseline HbA1c.

A proof‐of‐concept study, exploring the potential of the nocturnal glucose change measure, termed “BeAM”, as an indicator to intensify treatment targeting postprandial hyperglycaemia in people inadequately controlled on basal insulin, concluded that BeAM values between 2.5 and 3.1 mmol/L (45 and 55 mg/dL) should trigger the consideration of intensification.^11^ In our analysis, the median cut‐off was 3.1 mmol/L (56 mg/dL), which aligns with these observations, as only a modest improvement in HbA1c (−0.03%) was observed after 18 weeks' treatment with continued basal‐only treatment in participants with a nocturnal glucose change >3.1 mmol/L (56 mg/dL). Further, they reported that large BeAM values may correlate with higher incidence of hypoglycaemia.^11^ Similarly, the incidence of hypoglycaemia was numerically higher in participants treated with basal‐only insulin, with a nocturnal glucose change >3.1 mmol/L (56 mg/dL) compared with those with nocturnal glucose change readings under this cut‐off; therefore, the use of the nocturnal glucose change as an indicator for intensification may also help prevent the unnecessary uptitration of basal insulin and the corresponding increase in the risk of hypoglycaemia with no additional benefit in glycaemic control.

In addition to monitoring HbA1c, healthcare professionals should consider measuring nocturnal glucose change and/or PPG increment as these factors may provide additional information and, based on the preliminary findings here, appear to be predictive of positive glycaemic response to bolus intensification. Nocturnal glucose change is easier to obtain and calculate in clinical practice than PPG increment. Because of the correlation between PPG increment and nocturnal glucose change demonstrated in the present study, nocturnal glucose change might be a reasonable alternative to measuring PPG increments. This would be less inconvenient to the patient and of great value to physicians. Although nocturnal glucose change is easily calculated, a number of factors could influence the bedtime and pre‐breakfast values (eg, dinner meal composition, late‐night snacking, missed dose, nocturnal hypoglycaemia, quality of sleep); therefore, physicians would still need to instruct the patient on how best to obtain the values necessary to calculate nocturnal glucose change and maintain consistency in this technique as it is utilized to monitor dose titration.

There are some study limitations to consider. The analysis is exploratory in nature and based on one study alone. The findings are based on clinical trial data with faster aspart, a new prandial insulin analogue, on a background of three different basal insulins, and thus results may not be easily generalized. The influence of other factors on the bedtime and pre‐breakfast glucose measurements (listed above), and thus on the nocturnal glucose change value, was not analysed. Nevertheless, the findings reported in the present paper warrant further research into the use of nocturnal glucose as an accessible and clinically useful predictor for insulin intensification. Further studies would benefit from a standardized approach to measuring nocturnal glucose change, preferably using continuous glucose monitoring to capture the full range of glycaemic values while on treatment.

In conclusion, in the present trial population, the analysis suggests that PPG increment and nocturnal glucose change measurements may be more useful predictors than HbA1c for identifying people with T2D on basal insulin therapy, with or without oral antidiabetic drugs, who would most benefit from intensification with bolus insulin. Further, nocturnal glucose change may indeed perform just as well as PPG increment, while being easier to measure. The clinical applicability and potential use of nocturnal glucose change as a predictor for insulin intensification in T2D warrants further exploration.

## CONFLICTS OF INTEREST

A.L.P. has served on advisory boards for Abbott Diabetes Care, Becton Dickinson, Bigfoot, Eli Lilly and Company, Lexicon, Livongo, Mannkind, Medscape, Merck, Novo Nordisk, Omada Health, Sanofi and Zafgen, has served on a speakers' bureau for Novo Nordisk and received research funding from AstraZeneca, Dexcom and Mannkind. M.P. has participated in advisory panels for Novo Nordisk, Boehringer Ingelheim and Eli Lilly. J.E. is an employee of and holds stock in Novo Nordisk. K.S.‐S. is an employee of and holds shares in Novo Nordisk. J.S. is an employee of and holds stock in Novo Nordisk. K.B. has participated in advisory panels for AstraZeneca, Boehringer Ingelheim, Eli Lilly, Merck, Novo Nordisk, Sanofi, Johnson & Johnson, and speakers' bureau for Novo Nordisk and Sanofi.

## AUTHOR CONTRIBUTIONS

A.L.P. was the principal investigator. A.L.P. and K.B. are the guarantors of this work and, as such, had full access to all data in the study and take responsibility for the integrity of the data and accuracy of the data analysis. J.E. was the responsible statistician. All authors had access to the study data, take responsibility for the accuracy of the analysis, reviewed and contributed to the content of this manuscript, and had authority in the decision to submit this manuscript for publication, in collaboration with Novo Nordisk. All authors approved the manuscript for publication.

## Supporting information


**Figure S1** Onset 3 trial design.
**Figure S2**: Severe or BG‐confirmed hypoglycaemic episodes stratified by baseline parameters.
**Table S1**: Baseline characteristics at randomization of the onset 3 trial population.
**Appendix S1** Supporting Information

## Data Availability

The patient‐level analysis datasets for the research presented in the publication are available from the corresponding author on reasonable request.
