# Supplementary material for: Baseline nocturnal glucose change: A predictor of the treatment effect of bolus intensification in insulin‐treated type 2 diabetes
Source: Diabetes Obes Metab. 2019 Apr 23;21(7):1752–6. doi: 10.1111/dom.13729 (PMC6618272; doi:10.1111/dom.13729)
Supplement: Supplementary file 1 — Figure S1 Onset 3 trial design. Figure S2: Severe or BG‐confirmed hypoglycaemic episodes stratified by baseline parameters. Table S1: Baseline characteristics at randomization of the onset 3 trial population. Appendix S1 Supporting Information [file DOM-21-1752-s001.pdf]

## Supplementary Material

### Baseline nocturnal glucose change: a predictor of the treatment effect of bolus intensification in insulin-treated type 2 diabetes

Anne Peters, Milivoj Piletič, Johan Ejstrud, Karen Salvesen-Sykes, James Snyder, Keith Bowering

*Diabetes, Obesity & Metabolism*

### Supplementary Figure 1: onset 3 trial design

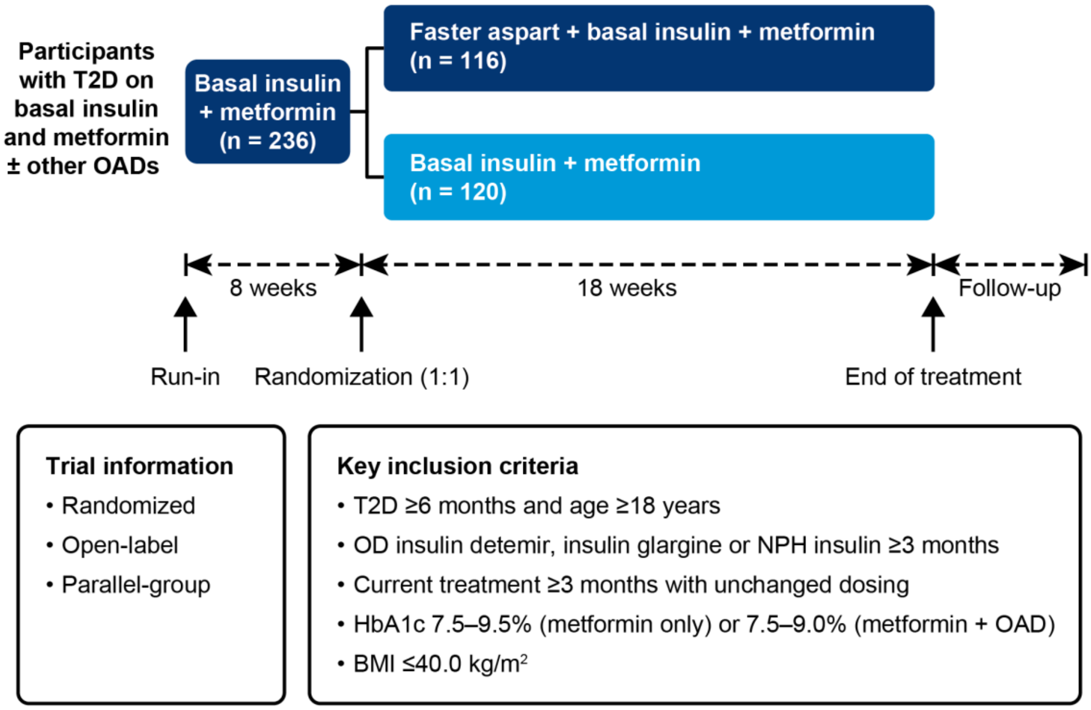


ClinicalTrials.gov: NCT01850615. BMI, body mass index; faster aspart, fast-acting insulin aspart; FU, follow-up; NPH, neutral protamine Hagedorn; OAD, oral antidiabetic drug; OD, once daily; T2D, type 2 diabetes.

**Supplementary Figure 2: Severe or BG-confirmed hypoglycaemic episodes stratified by baseline parameters**

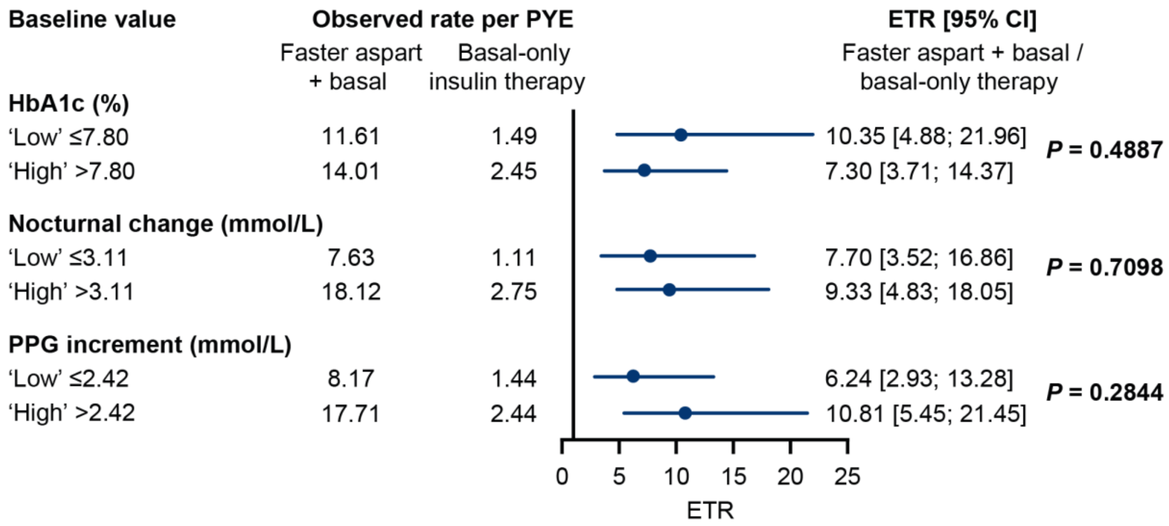


Severe or BG-confirmed hypoglycaemia: severe according to the ADA classification and/or recorded BG <3.1 mmol/L (56 mg/dL). ADA, American Diabetes Association; BG, blood glucose; CI, confidence interval; ETR, estimated treatment ratio; faster aspart, fast-acting insulin aspart; PPG, postprandial glucose; PYE, patient-year of exposure.

**Supplementary Table 1: Baseline characteristics at randomization of the onset 3 trial population**

| Characteristics, FAS | Faster aspart + basal (n = 116) | Basal insulin only (n = 120) | Total (n = 236) |
|---|---|---|---|
| **Age, years (SD)** | 57.5 (9.9) | 57.4 (8.5) | 57.4 (9.2) |
| **Gender, n (%)** | | | |
| Male | 55 (47.4) | 59 (49.2) | 114 (48.3) |
| Female | 61 (52.6) | 61 (50.8) | 122 (51.7) |
| **BMI, kg/m$^2$ (SD)** | 30.4 (5.0) | 31.1 (4.7) | 30.8 (4.8) |
| **Body weight, kg (SD)** | 82.2 (16.2) | 85.1 (17.3) | 83.7 (16.8) |
| **Duration of diabetes, years (SD)** | 10.9* (6.1) | 11.8 (7.4) | 11.3 (6.3) |
| **HbA1c** | | | |
| % (SD) | 7.9 (0.7) | 7.9 (0.7) | 7.9 (0.7) |
| mmol/mol (SD) | 63.2 (7.6) | 63.1 (7.4) | 63.1 (7.5) |
| **FPG** | | | |
| mmol/L (SD) | 7.4 (2.4) | 7.7$^\dagger$ (2.9) | 7.5 (2.6) |
| mg/dL (SD) | 132.5 (43.5) | 138.9 (51.4) | 135.7 (47.7) |
| **Basal insulin at baseline, n (%)** | | | |
| Insulin glargine | 76 (65.5) | 77 (64.2) | 153 (64.8) |

| | | | |
|---|---|---|---|
| Insulin detemir | 16 (13.8) | 17 (14.2) | 33 (14.0) |
| NPH | 24 (20.7) | 26 (21.7) | 50 (21.2) |

All values are arithmetic means, unless otherwise stated. *n = 115; †n = 119. BMI, body mass index; FAS, full analysis set; faster aspart, fast-acting insulin aspart; FPG, fasting plasma glucose; NPH, neutral protamine Hagedorn; SD, standard deviation.

Table reproduced and adapted with permission of John Wiley & Sons © 2017, from Rodbard *et al*. *Diabetes Obes Metab* 2017;19:1389–96.

**Supplementary Appendix**

*onset 3 trial: study design and insulin administration*

All participants required previous treatment with basal insulin to be included in the trial. The trial began with an 8-week run-in period during which metformin was continued, all other OADs discontinued, and basal doses were optimized using a treat-to-target approach, with a pre-breakfast SMBG target of 4.0–6.0 mmol/L (71–108 mg/dL). After the run-in period, the basal insulin dose was adjusted at the investigator's discretion. Basal insulin (100 U/mL) was injected subcutaneously once daily into the thigh or upper arm at approximately the same time every evening (NPH and insulin detemir using a 3 mL FlexPen; insulin glargine U100 using a 3 mL Solo-Star Pen). The chosen injection area remained the same throughout the trial, and injection sites were rotated within the same area.[1]

*onset 3 trial: baseline characteristics and insulin doses*

In the onset 3 trial, 236 participants were randomized to receive either faster aspart basal–bolus treatment (n = 116) or basal-only treatment (n = 120), both in combination with metformin. Baseline characteristics at randomization are reported in **Supplementary Table 1**. These were well matched across participants in both treatment arms. Of the total population, 48.3% were male (n = 114); the mean age, duration of diabetes, HbA1c and body mass index values at baseline were 57.4 years, 11.3 years, 7.9% (63.1 mmol/mol) and 30.8 kg/m$^2$, respectively. The mean dose of basal insulin in the group randomized to faster aspart and those who continued on basal insulin only post-randomization was 49.4 units (U) (0.6 U/kg) and 51.2 U (0.6 U/kg), respectively.[1] At the end of the trial (EOT), the mean basal and bolus insulin dose in the faster aspart group was 46.1 U (0.5 U/kg) and 55.6 U (0.7 U/kg), respectively, and in the basal-only group, the mean basal insulin dose was 55.9 U

(0.6 U/kg).[1] In the faster aspart group, mean body weight increased from baseline to EOT with an estimated treatment difference of 1.66 kg (95% CI: 0.89; 2.43; *P* < 0.0001).[1]

*Post hoc analysis: statistical methods*

Efficacy outcomes and correlations between baseline characteristics were calculated using the full analysis set; treatment rate ratios of hypoglycaemic episodes were calculated using the safety analysis set. The full analysis set included all randomized participants who received at least one post-baseline assessment. The safety analysis set included all participants who received at least one dose of the treatment drug. The correlation between baseline characteristics was determined using Pearson's correlation coefficients (r). Estimated treatment differences (ETDs) for the change from baseline in HbA1c and mean PPG increments at week 18 were analyzed using a mixed-effect repeated measures model (MMRM), including visit, treatment, region and previous basal insulin usage as fixed factors, participant as random effect, baseline value of the endpoint (HbA1c or mean PPG increment) as covariate, and the interaction between visit and all fixed effects and covariates. Treatment rate ratios were estimated with a negative binomial regression model, using a log-link function and the logarithm of the time for which a hypoglycaemic episode was considered treatment emergent as offset. The model included region and previous insulin usage as covariates and interactions and main effects between treatment and the subgroup. The *P*-values for homogeneity of treatment differences between 'high' and 'low' subgroups were calculated with an F-test for change from baseline in HbA1c and mean PPG increments at week 18, and a Wald test for the rate of hypoglycaemia. Statistical significance was defined as *P* < 0.05.

**Reference**

1. Rodbard HW, Tripathy D, Vidrio Velazquez M, Demissie M, Tamer SC, Piletic M. Adding fast-acting insulin aspart to basal insulin significantly improved glycaemic control in patients with type 2 diabetes: a randomized, 18-week, open-label, phase 3 trial (onset 3). *Diabetes Obes Metab* 2017;19:1389–1396.
